# Compounding Local Invariant Features and Global Deformable Geometry for Medical Image Registration

**DOI:** 10.1371/journal.pone.0105815

**Published:** 2014-08-28

**Authors:** Jianhua Zhang, Lei Chen, Xiaoyan Wang, Zhongzhao Teng, Adam J. Brown, Jonathan H. Gillard, Qiu Guan, Shengyong Chen

**Affiliations:** 1 College of Computer Science, Zhejiang University of Technology, Hangzhou, Zhejiang, China; 2 Department of Radiology, University of Cambridge, Cambridge, United Kingdom; 3 Division of Cardiovascular Medicine, University of Cambridge, Cambridge, United Kingdom; Institute of Automation, Chinese Academy of Sciences, China

## Abstract

Using deformable models to register medical images can result in problems of initialization of deformable models and robustness and accuracy of matching of inter-subject anatomical variability. To tackle these problems, a novel model is proposed in this paper by compounding local invariant features and global deformable geometry. This model has four steps. First, a set of highly-repeatable and highly-robust local invariant features, called Key Features Model (KFM), are extracted by an effective matching strategy. Second, local features can be matched more accurately through the KFM for the purpose of initializing a global deformable model. Third, the positional relationship between the KFM and the global deformable model can be used to precisely pinpoint all landmarks after initialization. And fourth, the final pose of the global deformable model is determined by an iterative process with a lower time cost. Through the practical experiments, the paper finds three important conclusions. First, it proves that the KFM can detect the matching feature points well. Second, the precision of landmark locations adjusted by the modeled relationship between KFM and global deformable model is greatly improved. Third, regarding the fitting accuracy and efficiency, by observation from the practical experiments, it is found that the proposed method can improve 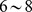
% of the fitting accuracy and reduce around 50% of the computational time compared with state-of-the-art methods.

## Introduction

The majority of medical image processing tasks, including fMRI analysis [1], 3D reconstruction [2] [3], and medical image segmentation [4] [5] [6], rely heavily on accurate image co-registration. To date, many methods exist that can help perform image co-registration (e.g. [7] [8] [9] [10] [11]). A comprehensive review summarizing these methodologies has been performed in [12], which divides them into two main categories; geometric image features-based and voxel similarity measures-based. Among these registration methods, active contour models (ACM) [13], active shape models (ASM) [14] and active appearance models (AAM) [15] are particularly important methods. They are widely employed and have greatly improved medical image analysis, due to their excellent non-rigid deformable performance [16] [17] [18] [19].

However, there are still several critical issues to be resolved. Two recent surveys, [20] and [21], summarized the deformable model and medical image segmentation respectively, and identified problems, such as model initialization, and the robustness and accuracy for inter-subject anatomical variability, both of which need to be improved.

The results of deformable model rely heavily on its initial position. If the initial position of the model is close to the region of interest, fitting results will be more accurate and the evolution time will be shorter. On the other hand, if the initial position is far away, it can lead to failure, due to the limited local searching algorithm of deformable models. Therefore, it may be worth investigating how to guarantee that the initial model is within the searching range. However, most attempts at improving deformable models have ignored this problem and left users to manually initialize it. Few research projects involve automatic initialization, and are limited to special applications ([22] [23] [24]) or unique deformable models ([25] [26]). Garrido and Qerez proposed a reformulated Hough transform to initialize the deformable model for cell segmentation [22], but there were some cells which could not be correctly found in the image, and the algorithm did not consider the performance of initialization when the target image was deformed. In [23], the authors implemented a fast 3D generalized Hough transform based on prior information of heart position, which is designed for a cardiac model. Wang et al. [24] proposed to use irises to initialize ASM for use in facial recognition. The initialization presented in [25] is limited to ACM and can not be extended to ASM or AAM. In [26], the initializing method is constrained to an improved ASM, resulting in an exhaustively search of a whole image to obtain good initialization. In [27], a method of automatically initializing a 3D deformable model is presented by compounding a pictorial structures model [28] and AAM. However, features used in this method are less distinctive than that used in our model. The method proposed in [29] only used Hough transform to determine the initializing model by a bounding box, where only rough positions of each landmarks can be located.

Inter-subject anatomical variability may abolish one-to-one image correspondence between subjects. Thus global deformable geometrical model will not fit objects well, particularly when the same anatomical structures are absent in different subjects. Only a few methods have investigated this problem. [30] improved the AAM through outlier detection techniques. Toews et al. also employed local invariant features to train a statistical part-based model [31]. This model learns the probability of a feature presence (or absence) from a large image set and matches local invariant features to a new image allowing fitting. The advantage of this model is that it does not rely on exact anatomical similarity between subjects to permit co-registration, as it will typically locate some corresponding local structures. However, the methodology used ensures that the images produced may not have clear anatomical meanings.

In an attempt to address the problems mentioned above, we propose a novel compounding model by combining a deformable geometric model and local invariant features. Local invariant features can extract local structures robustly and consequently match them more accurately to images from different subjects. In this paper, we exploit the scale invariant feature transform (SIFT) [32] to construct our compounding model. It is worth noting that the proposed model can be easily extended to other local invariant features. We first build the deformable geometric model, which is the point distribution model (PDM) in this study, by manually labeling landmarks and aligning the shape represented by landmarks in all images of the training set. There are two stages to choose landmarks. At the first stage, we choose those key points that correspond to anatomic structures and some special structures, such as T-junction. At the second stage, we uniformly choose points between two adjacent landmarks selected in the previous stage. Then, we extract the SIFT features from the training images and align them to the PDM. Two PDMs used in our experiments both have 38 landmarks, in which 8 are key points and 30 are other landmarks uniformly selected. We have developed a strategy to preserve the highly-repeatable and highly-robust SIFT features. Based on these selected SIFT features, an efficient and automatic learning strategy is developed to build a Key Features Model (KFM). The KFM is then connected to the PDM by modeling their geometrical relationship. Based on this relationship, we develop an automatic strategy, precisely initializing the position of PDM. After the initialization of PDM, more precise localization of each landmark is also achieved. Finally, the ultimate positions of landmarks are obtained by iterative search processes.

Unlike other initialization methods mentioned previously, the proposed method can initialize global deformable geometrical models in more extensive ranges, due to its accuracy in matching local parts. Unlike Toews's model, the proposed model connects the local parts with explicit anatomical structures represented by PDM. Therefore the proposed method can find local parts among variable inter-subject anatomical tissues, as well as the meaningful anatomical structures.

Extensively experiments have been carried out to evaluate the performance of the proposed method. We have collected data from a large number of cardiac MRI images, consisting of 500 slices from 16 subjects, 10 male and 6 female, aged from 45–64 years. Firstly, we demonstrate the matching accuracy of the proposed KFM with respect to its repeatability and robustness. Then the accuracy of initialization and fitting is evaluated. Finally, our fitting results show the 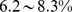 improvements by comparison with an improved ASM [16].

The main conclusions of this paper is three-fold: 1) A KFM is built to match repeatable local parts among subjects with anatomical variance, even when no exact one-to-one correspondence exists. 2) An automatically initializing strategy is developed for the initialization of PDM. 3) An efficient strategy is developed to precisely localize landmarks of PDM.

## Methods

### Ethics statement

This study was approved by the Research Ethics Committee of Zhejiang University of Technology and Hangzhou Shaoyifu Hospital, China. All subjects used in this paper have been informed that their data may be published and provided written informed consent.

### Model building

The new model consists of a PDM, a KFM and their geometric relationship. The PDM is built in an ASM first and the gray-levels of pixels around the landmarks in the PDM are also modeled. The KFM is built by extracting SIFT features and choosing high-repeatable and high-robust ones. This section explains how to build PDMs and KFMs, and model their geometric relationship.

#### Point distribution model

A PDM consists of 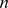 landmarks that are the important points to represent the particular parts or boundary of objects in the training set. The collection of these landmarks is used to define a shape vector as 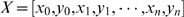. These shapes in all training images must be aligned with respect to a set of axes. The required alignment is achieved by scaling, rotating, and translating the training shapes so that they agree as closely as possible. From all aligned shapes, a mean shape is computed by 
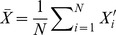
, where 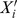 indicates an aligned shape. Shape dimensions and variations are reduced by using the Principal Components Analysis (PCA). Thus a new shape is obtained by:
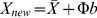
(1)where 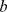 is a 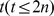 dimensional vector containing the PDM's parameters, and computed by:



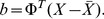
(2)


A new shape 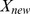 is obtained by varying 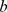 within a constrained range 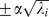, where 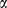 is usually set to a value between two and three. And the new shape should be transformed into a new and better location on the target image.

To find the accurate position of the new shape, the gray-level appearance should also be modeled. For each landmark, gray-level values are drawn from a line. This line passes through the landmark and is perpendicular to the boundary, which is formed by the landmark and its neighboring pixels. For *j*-th landmark in the *i*-th image, 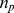 extracted gray-level values centered at *j*-th landmark form a gray-level profile, denoted by 
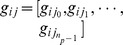
. Thus a mean profile is defined as:
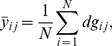
(3)where 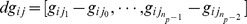. The final new shape is obtained by finding such a shape that the profiles of landmarks in new image have minimal difference with respect to their mean profiles and its variations do not exceed the range of 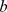.

#### Key features model

Given total 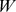 images in the training set, 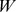 groups of SIFT features need to be extracted. However, there are too many SIFT features, even if there are only a few objects in one image. These features should be selected carefully and only those highly-robust and highly-repeatable key points can be used to establish the KFM. There are three steps to choose the suitable SIFT features.

The first step is to align all 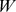 groups of SIFT features to the mean shape obtained when building the PDM. When building the PDM, we align shapes on different training images as closely as possible. This procedure provides a set of transforming parameters. By applying these parameters to corresponding SIFT features, they can also be aligned to the mean shape of the PDM. If SIFT features in different images are associated with the same position, they will be closely located after being aligned.

**Figure 1:**
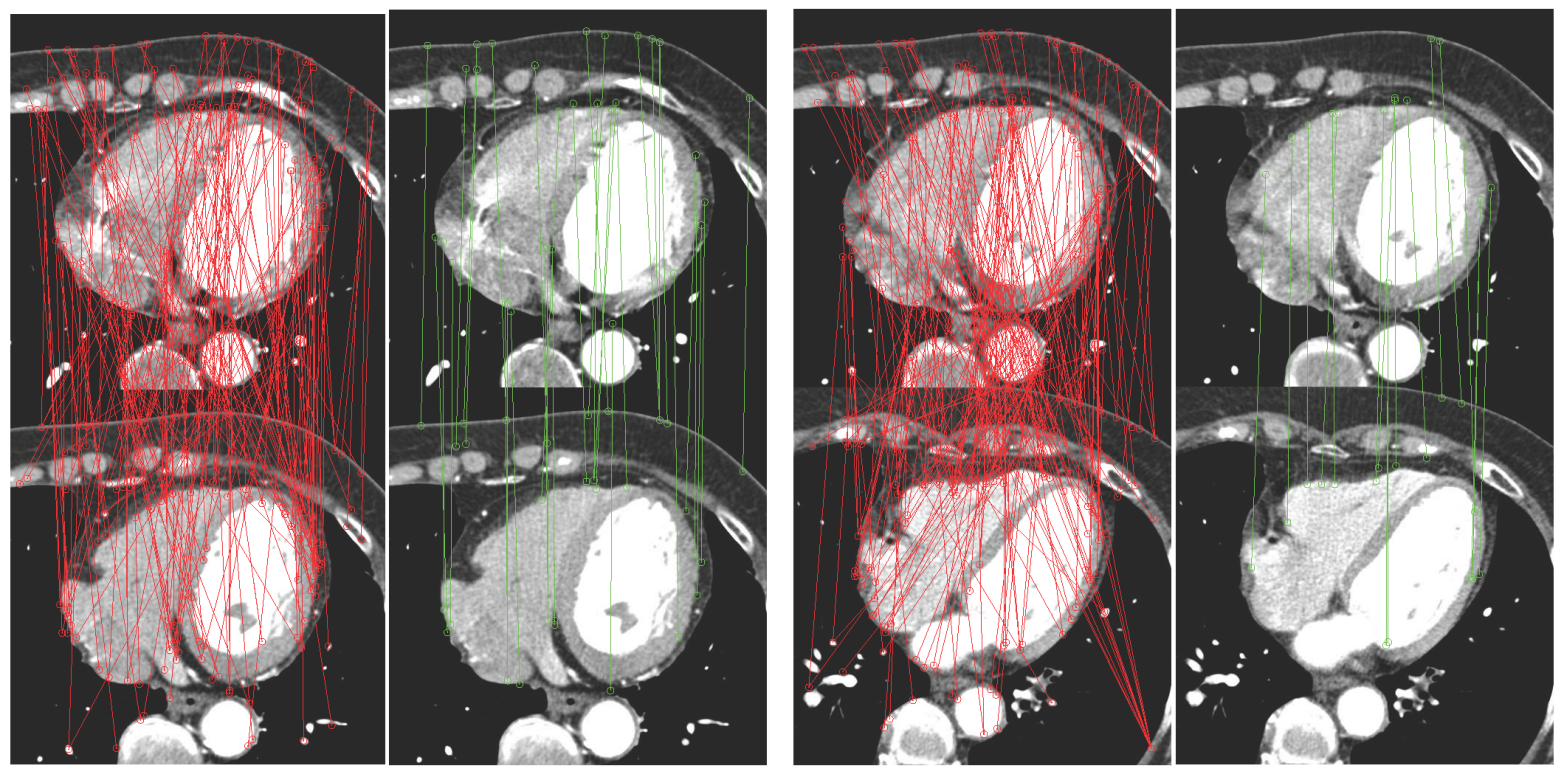
Two examples of matching results of the SIFT features. Picture marked by red points and lines illustrates the results of using only previous two conditions. Pictures marked by green points and lines illustrates the results of using three conditions.

The second step is to match the SIFT features to obtain highly-robust matching pairs. Usually, the appearance description of SIFT features is used to match SIFT features. SIFT features are local invariant features and their descriptions only represent their local image information. It may result in mismatched pairs, if there are similar local structures in two image but at different positions. After aligning SIFT features, an extra geometric constraint can be used to improve the matching correctness. Assuming two images in the training set are denoted as 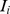 and 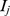 and their SIFT features are denoted as 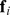 and 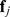, respectively. For one SIFT feature in the image 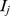, 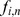, its matched SIFT feature in the image 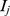 can then be determined if they meet the following conditions:
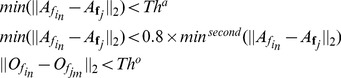
(4)where 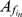 and 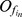 denote the appearance description and aligned geometric position of 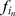. The previous two conditions, suggested in [32], are usually used for SIFT feature matching. Here an extra condition based on aligned SIFT features is added. Assuming 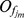 and 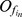 are the aligned geometric position of 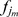 and 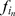, if they meet the previous two conditions, they are considered as the potential matching pair. If the geometric distance between 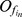 and 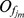 is less than a threshold, they are regarded as a matching pair. Some examples is shown in Fig. 1, from which we can observe that the third condition will greatly improve the matching accuracy.

By using these conditions, SIFT features of each image are matched to all other images in the training set and a set of matched pairs can be obtained. We denote 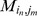 as the *n*th SIFT feature in the *i*th image being matched to the *m*th SIFT feature in the *j*th image.

The third step is to choose highly-repeatable SIFT features by a matching propagation procedure. Given a SIFT feature, 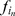, we will choose all SIFT features corresponding to the same position in different images to form a collection 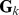. Firstly we initialize 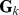 by adding 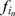 into it. And we choose all SIFT features in other images that match with 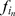 to be members of 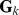. For each SIFT feature in 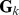, we then choose all SIFT features in the other image that matches with it and then add them into 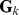; repeating this procedure, until there are no other SIFT features in all images that are matched with any features in 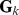. Thus a collection of SIFT features in all images corresponding to the same position as 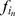 can be obtained. Performing this procedure is necessary and important. One SIFT feature in one image may not find matched features from other training images. When applying matching propagation, we can obtain as many SIFT features as possible, corresponding to the same position in all images of the training set.

**Figure 2:**
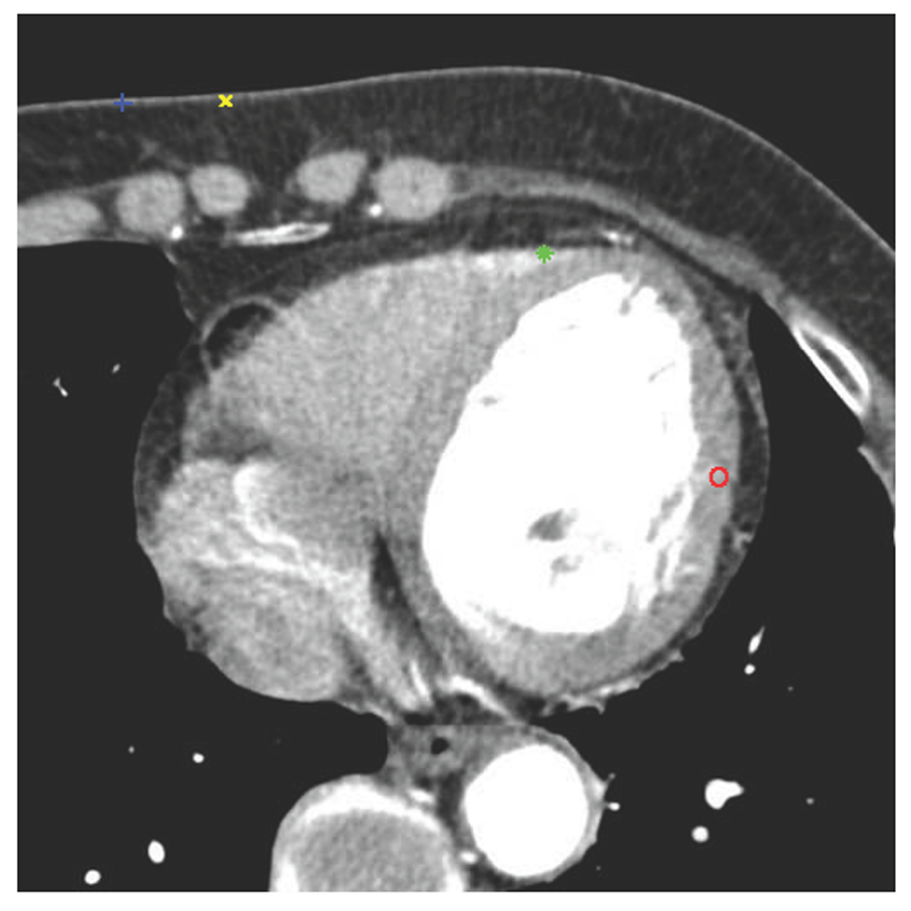
Features in one slice with different occurrence ratios. The ratios of red, green, blue and yellow features are 0.85, 0.73, 0.52 and 0.31, respectively.

Supposing we finally obtain 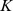 collections and each collection has 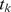 SIFT features after the above matching propagation procedure. Since there is only one SIFT feature associated with one position in one image, 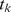 can reflect the number of images, in which robust SIFT features can be found at a certain similar position. The larger value 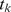 is, the higher repeatability the collection 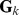 is. To guarantee high-repeatability, the ratio between 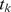 and the total number of training images must be larger than a constant value 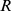. 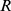 can be adjusted to balance the number of key points in KFM and the repeatability of the picked SIFT features. If 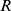 is set to a larger value, the number of key points will be decreased and the repeatability will be increased, and vice versa. In this study, according to the experiments in the sub-section of Evaluation of Different Picking Ratios, we suggest an adequate ratio (e.g. 0.6). An example is shown in Fig. 2, where the features with higher occurrence ratios are more likely located at meaningful positions, such as red and green feature points.

According to the three steps detailed above, the highly-robust and highly-repeatable SIFT features are obtained effectively. We can then use these SIFT features to build a KFM. A KFM consists of 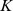 sets of SIFT features. In each set, SIFT features locate at the close positions. The average appearance description of each SIFT features set, 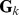, is computed as:
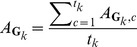
(5)where 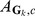 means the appearance description of the *c*th SIFT features in 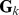.

If a SIFT feature, 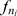, in a test image meets the following conditions simultaneously, we consider it match to a collection 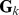 in KFM:
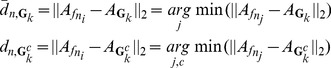
(6)where 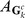 is the appearance description of the *c*-th SIFT feature in 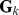. Thus the first equation in (6) means 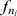 has the most similar appearance description with 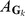 with respect to other SIFT features in the test image. The second equation in (6) means the minimal distance between 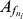 and appearance descriptions of SIFT features in 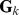 is also the minimal distance between the appearance descriptions of any SIFT features in the test images and appearance descriptions of SIFT features in 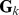.

#### Statistical geometric relationship between KFM and PDM

The relationship between the KFM and the PDM is built through relative displacements between aligned positions of SIFT features in the KFM and aligned landmarks in the PDM. We denote the geometric position of the *c*th SIFT feature in the *k*th SIFT feature set of KFM as 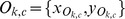 and the position of the *l*th landmark in PDM as 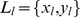. The displacement between 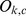 and 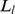 is then denoted as 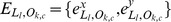. From the statistical perspective, given one landmark 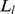, we have:
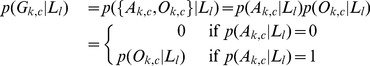
(7)


Given a training image, the SIFT feature 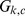 may be present or not, therefore the probability 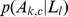 is equal to 1 or 0. The position of this SIFT feature can be denoted by its displacement relative to the given landmark 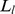, and therefore we have:
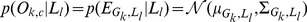
(8)where the displacement of one SIFT feature in one set 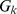 is assumed to be subjected to bivariate Gaussian distribution, and 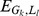 denote the displacement between 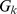 and landmark 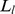. Given a test image, in which the positions of landmarks are unknown, if the *u*-th SIFT feature 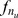 in this image is found to match to one SIFT feature in 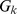, the matched SIFT features can be used to determine positions of landmarks.
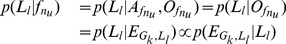
(9)


Because in (8) positions of SIFT features in 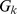 can be determined by its displacement relative to the *t*th landmark, the position of this landmark in a test image can accordingly be determined by its displacement relative to the matched SIFT feature.

For each set of SIFT features in KFM, there are the relations denoted by (8) and (9), the position of the landmark in a test image is therefore determined by all SIFT features each of which is matched to one of the SIFT feature sets. It is worth noting that there may not be a matched SIFT feature for every 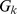. Finally, the position of the landmark is calculated by maximizing the joint distribution determined by all observed matched SIFT features:
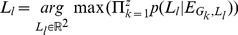
(10)where we assume 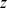 SIFT features, each of which matched to one 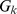, are found. By using the logarithm of (10) and solving its extrema, it is possible to obtain the position of landmarks.

#### Extend to other local invariant features and deformable models

In the proposed model, we only assess the appearance description and geometric position of local invariant features. However, it does not depend on special local invariant features. Therefore the proposed model can be easily integrated with other local invariant features, such as the maximally stable extremal regions (MSER) [33] and the speeded up robust features (SURF) [34]. In the matching propagation steps mentioned in the sub-section of Key Features Model the appearance description of the SIFT features can be replaced by another correspondence description and their matching algorithm can also be followed with an extra geometric constraint.

The proposed model can also be extended to the other deformable models with little difficulty. What the proposed model needs is a reference framework, just like the manually labeled landmarks in ASM. By using the reference framework, those extracted local invariant features can be normalized and the extra geometric constraint can be applied to improve the matching correctness. The AAM is similar to ASM and naturally has a reference framework since it also contains a manual labeling step. If using AAM instead of ASM in the proposed model, we can follow all the steps detailed previously. For ACM, an extra learning procedure is required. In this learning procedure, several reference points need to be labeled to normalize the local invariant features and the relationship between reference points and local invariant features will also be learned. The initial position of ACM can then be determined by the relationship between those matched local invariant features and interesting objects.

### Fitting process

#### Initialization

For initializing the model in a test image, the SIFT features are extracted first and matched to the KFM. The first two matching conditions in (4) are used, which are similar to the strategies suggested in [32]. Due to the highly robust SIFT feature sets in the KFM, there are only a few mismatching points. We employed the method proposed in [35] to eliminate the mismatching SIFT feature pairs. After obtaining the accurately matched SIFT feature pairs, we can compute transformation parameters, 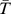, 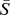 and 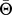 that transform the average positions of SIFT features in the KFM towards the positions of SIFT features in the test image. Thus, by applying these parameters to the mean shape of PDM, we can initialize the PDM model to an appropriate position:
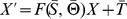
(11)where 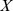 is the mean shape in the PDM and 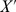 is the initialized model.

#### Accurately localized landmarks

After obtaining an initialized model 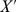, each landmark in 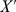 is still located at its rough position, as the transformation in the previous subsection is a type of rigid transformation that maintains the original contour of mean shape 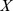. The position of each landmark can be modified according to the relationship between the KFM and the PDM built in the sub-section of Iterative Process to obtain the precise position.

Assuming there are 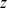 matched SIFT features in a new image, the position of each landmark can be determined by (11). According to the relationship between the KFM and the PDM, we can further improve landmark positions by using (10). Then this landmark will move towards this new position from the old position in 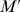. The PDM, however, is a global constrained model and the modification of the position of one landmark will influence other landmarks' positions, which is very important to keep the whole model from being abnormal. The positions are modified according to (12), which is similar to the elastic model. That is to say, if other landmarks are closer to one landmark, the forcing movement of these landmarks is larger than that of landmarks further away. Assuming the new position of the 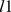
th landmark is 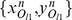 and its old position in 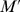 is 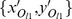, the modified position of the 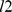
th landmark caused by the movement of the 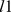
th landmark can be computed by:
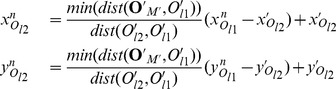
(12)


where 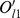 means the position of the 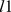
th landmark in the initialized PDM. 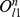 is the precise location of this point. 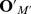 is the points set in PDM and 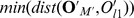 means the minimal distance between 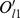 and other points in PDM. The distance between 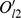 and 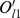 is denoted as 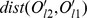. 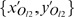 is the coordinate of point 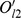. Hence, 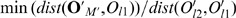 means the ratio of distance between the 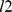
th landmark and 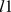
th landmark related to the minimal distance between other landmarks and 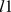
th landmarks. Therefore, when 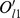 is modified to a precise pose, it will influence other landmarks' positions. Thus, more accurate location for the whole PDM can be obtained after pinpointing all landmarks.

#### Iteratively fitting process

After the precise position of each landmark in the PDM is computed, the same iterative process to ASM can be used to obtain the ultimate position. After obtaining a new shape 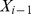 in the 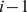
th iterative step, the *i*th new shape is computed by the following steps:

1. For one landmark, e.g. the *j*th landmark, in 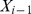, we extract 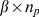 points' gray-level values to compute gray profile 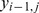, where 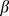 is set to 2–5;
2. Then we compute the difference between the mean profile 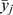, obtained by (3) in the training procedure, and any continuous 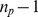 items in 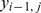 to find one point centered at which the difference between continuous 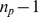 items and 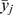 is minimum;
3. Repeating the pervious two steps and obtaining a new set of points, denoted by 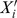;
4. Next we transform 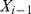 to 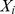 according to (1) to make sure that 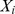 is closest to 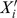.

These iterative steps usually are time-consumed and need a hundred iterations to converge if the initial position of PDM is not ideal. By applying the proposed method, however, the number of iterations will significantly decrease because each landmark was located at its precise position. Thus the time cost will be sharply reduced.

## Experiments

We evaluate the proposed method in a set of cardiac MRI images. Experiments have been carried out from three different aspects. First, we tested the repetition and robustness of the KFM, which is the base for the proposed model, determining the accuracy of initialization and fitting results. Second, the accuracy of initializing PDMs was evaluated. The accurate initialization will decrease iterative times and reduce the computational cost. Finally, we evaluated the speed and accuracy of co-registering cardiac images. In this study, the SIFT implementation used in our experiments is publicly available in http://www.cs.ubc.ca/~lowe/keypoints/.

### Datasets

**Figure 3:**
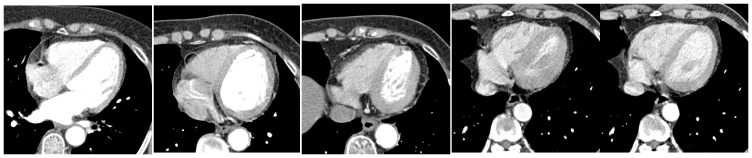
Some examples of cardiac MRI images used in our experiments.

A large number of cardiac MRI images were collected for the experiments. The data consisted of 200 slices from 16 subjects, 10 male and 6 female, aged from 45–64 years. Five groups of experiments were carried out based on five randomly selected image sets. In each group, we randomly select 10 subjects and approximately 120 slices. Around 60 slices from 5 subjects were used for model training, and the remainder for testing. Examples of the experimental images are shown in Fig. 3.

### Matching repeatability and robustness of KFM

**Figure 4:**
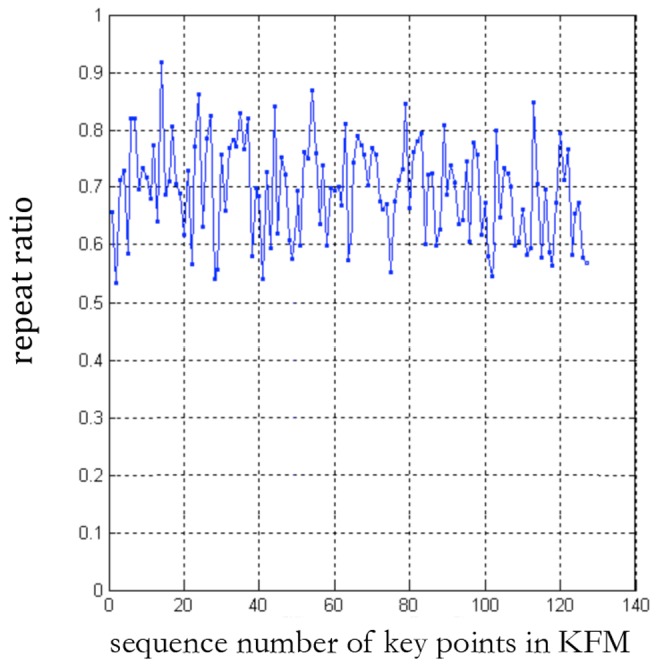
Repetition Ratio of all Key Points in KFMs.

As the proposed model is based on the highly-repeatable and highly-robust key points in the KFM, the repetition and robustness of the KFM are investigated first. In five groups of experiments, the numbers of key points in each KFM are 28, 26, 31, 20, and 22 respectively. To evaluate the repeatability of the KFM, the SIFT features were extracted from all images tested, and these features matched to the corresponding KFM. The number of times that correctly matching key points in KFM with test images were accumulated, allowing calculation of the repeated ratio for these key points. The minimum repetition ratio was 0.53 and the maximum repetition ratio was 0.96. The repetition ratios of 127 key points in all five KFMs are shown in Fig. 4, proving that enough repetitions of the KFM could be obtained.

Additionally, we calculated the maximum, minimum and average numbers of matching SIFT features in a new image. Table 1 illustrates these numbers for the five experimental groups. The number of matching SIFT features for each test image ranged from 10 to 22, with an average of 18.2 in the first experimental group. This means that at least 10 exactly matched SIFT features could be found in a new image within the first experimental group. According to these matched SIFT features, we can accurately initialize the PDM in accordance with the proposed initializing strategy.

**Table 1:** Number of matching SIFT features.

Group index	Maximum number	Minimum number	Average number
1	22	10	18.2
2	23	8	16.8
3	25	13	20.6
4	16	6	12.5
5	16	8	11.9

**Figure 5:**
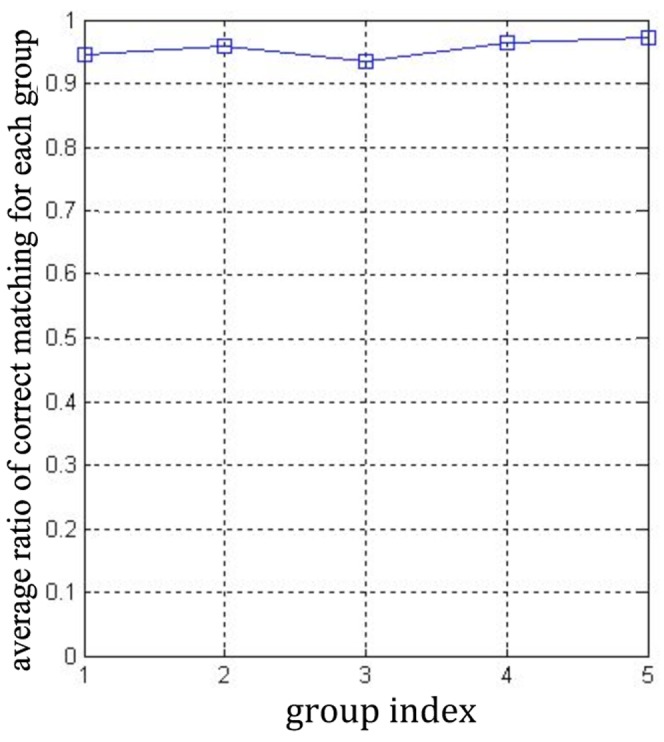
Average ratio of correct matching of all five groups of KFM.

For further evaluating the matching correctness of KFM, we compute the average ratio of correct matching, which is defined as the ratio of correct matching points to all matching points for all test images. In all five KFMs used in the experiments, the maximum and minimum average ratios of correct matching are 1 and 0.91, respectively. We illustrate the average ratios in Fig. 5, which demonstrate the high-robustness of KFM.

From Table 1, we observe that we can obtain enough matching key points when using the KFM to match SIFT features extracted from a medical image. SIFT features can achieve good performance of matching the same object at different images in a natural scenario. However, because there are lots of duplicated local structures and flexible deformation of hearts in different patients' MRI images, most SIFT features are not as robust as obtained in natural images. But through the proposed KFM, the most robust SIFT features can be identified and matched accurately. As shown in Fig. 5, the accuracy of matching results using KFM is at the same level as that of matching objects in nature scenarios with SIFT features. Therefore, our KFM can represent a powerful tool to find accurately matched points in different patients' cardiac images. From Fig. 4 and Fig. 5, it is obvious that each key point in the KFM has a high matching accuracy with considerable repeatability.

Toews's model also matches features extracted from the new image [31]. However, it has to match all SIFT features extracted from all training images, which takes a much longer time. Furthermore, since all SIFT features extracted from training images are used, more matching errors may be generated. By comparison with Towe's model, the proposed KFM only consists of selected highly-repeatable and highly-robustly local invariant features, and has potential to reduce the computational power required while improving matching accuracy.

### Evaluation of different picking ratios

As mentioned in the sub-section of Key Features Model, the picking ratio is an important parameter that balances the repetition and amount of key points in the KFM. It must be tested carefully and only the most proper ratio can be adopted. We evaluate several different picking ratios, such as 0.5, 0.6, 0.7 0.75, 0.8, 0.85 and 0.9. The number of key points and the repeated ratio under different picking ratios were computed respectively. All experiments in this section are based on the image dataset from the first experimental group.

**Figure 6:**
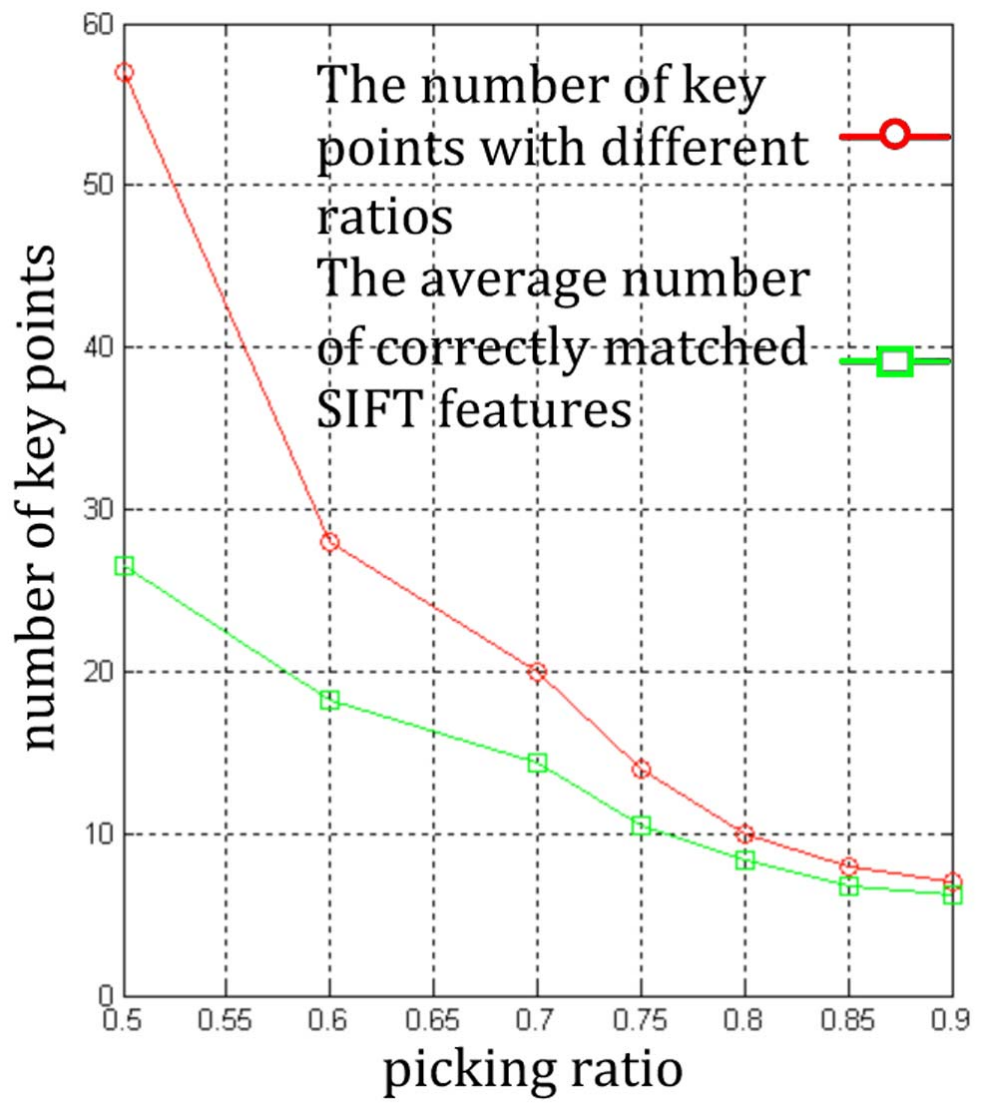
Number of key points in KFM and SIFT features under different picking ratios.

**Figure 7:**
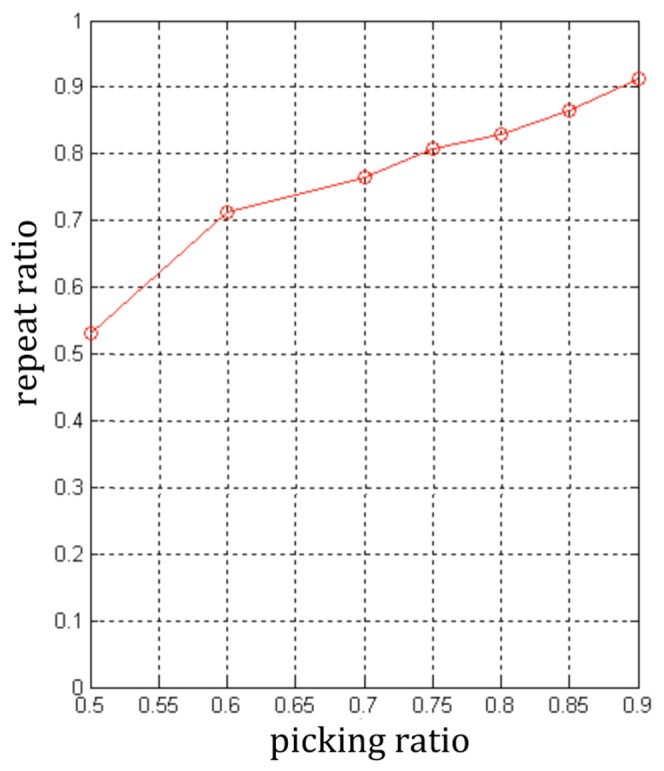
Repetition ratio under different picking ratios.

**Figure 8:**
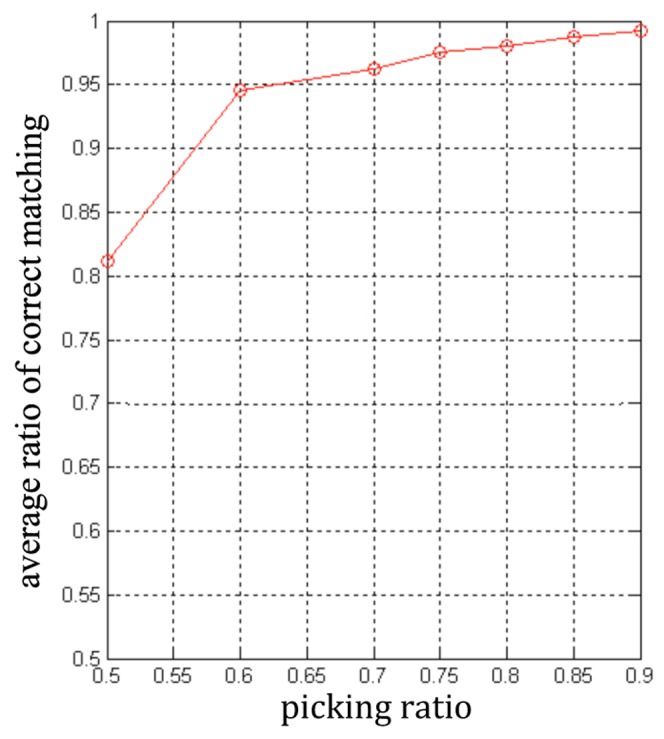
Average ratio of correct matching under different picking ratios.

Fig. 6 illustrates the number of key points under different ratios in KPMs and Fig. 7 shows the repeated ratio of key points under different picking ratios. The average number of matching SIFT features in the test image under different picking ratios is shown in Fig. 8.

In Fig. 6, when the ratio rises from 0.5 to 0.6, the number of key points sharply decreases, but the decrease of the average number of matching SIFT features is less. When the ratio keep increasing, the key points' amount and the average number are gradually approaching. This figure implies that the KFMs with low ratio have low-repeatable key points. Moreover, the low ratio implies the low repeatability and low accuracy of matching results. Fig. 7 and Fig. 8 illustrate these results.

From Fig. 7 and Fig. 8, when the ratio is far below 0.6, the repeatability and correct matching rate is too low to ensure that there are enough correct matching SIFT features in the new image. If the number of correctly matching SIFT features is not enough, the initialization will fail. On the other hand, when the ratio is much larger than 0.6, the accuracy of matching can be high enough, but the number of matching SIFT features is too low. Only when the ratio is set to about 0.6, both the repeatability and the matching accuracy achieves a good level.

### Accuracy of automatic initialization

As the first step of the fitting process, the initialization is very important. The accuracy of initialization directly affects the fitting results. In the standard ASM, there is no strategy to accurately and automatically initialize the PDM. In our experiments, we achieve enough matched key points with a high enough accuracy to initialize the PDM by using the KFM. For evaluating the accuracy of initialization, we employ the average error which is defined in [24] to calculate the average distance between the manually labeled shapes and the initialization results.
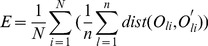
(13)where 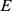 is the average error, 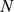 is the number of test images in one group of experiments, and 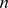 is the number of landmarks. 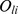 is the *l*th landmark point in the manually labeled shape of the *i*th test image manually labeled, 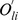 is the *l*th landmark point in the resulting shape of initialization for the 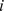
th test image.

The ratio of correct initialization is defined as the ration between correct initialization of the PDM and the number of all test images. The correct initialization of the PDM is determined manually. Actually, only these initialized PDMs are treated as correct initialization, if the number of landmarks, whose distance to the corresponding manually labeled landmarks is less than five pixels, is more than half of landmarks in the PDM. Table 2 shows the average accuracy of initialization.

**Table 2:** Accuracy of initial position.

Group index	Ratio of correct initialization	Average error
1	0.982	2.98
2	0.976	3.06
3	0.968	3.15
4	0.973	3.04
5	0.965	3.16

### Precision of pinpointing landmarks

During the process of pinpointing landmarks, the evaluation of precision is dependent on the distance between the position of manually labeling landmarks and pinpointing results by the proposed method. If the distance is less than three pixels, we believe that a landmark is precisely pinpointed. In all five experimental groups, the average number of precisely locating landmarks and the average precision are shown in Table 3. We also employ (13) to compute the average precision. In all models, there were 38 landmarks. From Table 3, the maximal and minimal numbers of precise pinpointing are 35 and 17, respectively. The average numbers are all more than 25. To the whole model with 38 landmarks, these precisely located landmarks are enough to adjust the whole model to a more accurate position by (12). By comparing with the initialized PDM, we found that the distance between the new position of the PDM and the manually labeled landmarks is smaller. As shown in Tabel 3, the reduced average errors with respect to the average errors obtained in the previous experiments are ranged from 0.3 to 0.7, which demonstrates that the process of pinpointing landmarks is effective.

**Table 3:** Evaluation of pinpointing landmarks.

Group index	Max number	Min number	Average number	Average error
1	34	18	28.5	2.56
2	30	18	27.3	2.34
3	33	17	26.2	2.83
4	30	20	25.1	2.67
5	35	21	29.8	2.72

### Accuracy of fitting results

We now evaluate the accuracy of the fitting results of our model by comparison to an improved ASM proposed in [16]. Table 4 shows their fitting results, in which the accuracy is computed by (13).

**Table 4:** Performance comparison between an improved ASM and our model.

Group index	Average error of [6]	Average error of our model	Improvement
1	2.16	1.98	8.3%
2	2.19	2.03	7.3%
3	2.23	2.09	6.2%
4	2.07	1.93	6.7%
5	2.11	1.95	7.6%

**Figure 9:**
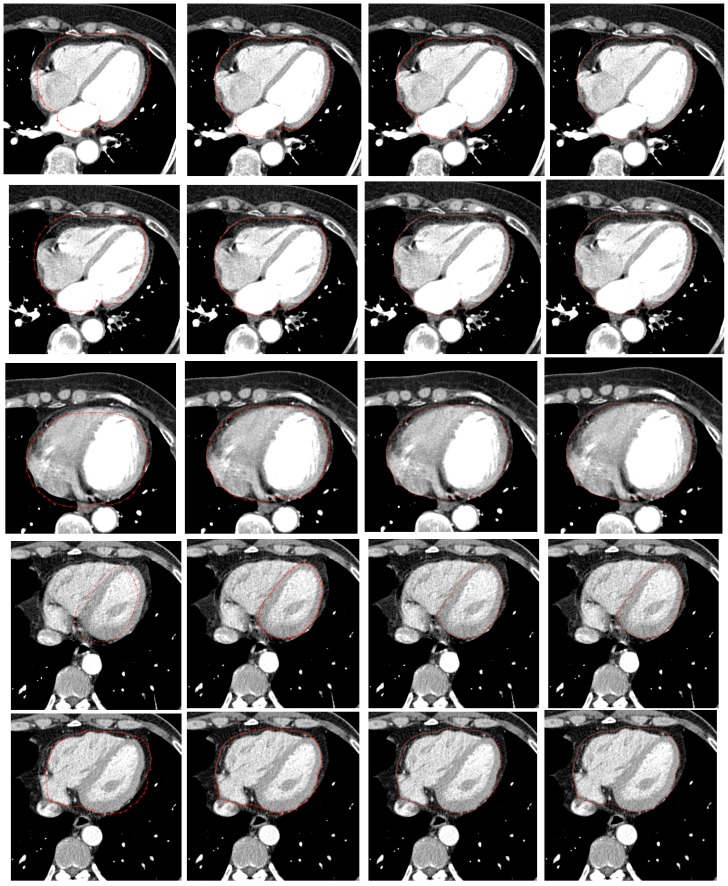
Some fitting results of our model. (Left) Pre-initialized model;(Middle left) Initialized model; (Middle right) Pinpointed model, note that there are some points that have been accurately located; (Right) Fitting result.

Firstly, as Table 4 shows, the average fitting errors resulting from our model are less than the improved ASM in [16]. The improvement of automatic initialization ranges from 6.2% to 8.3%. This proves that our proposed model can achieve more accurate results than that of the improved ASM. Furthermore, due to accurate initialization and precise pinpointing, it will also decrease the running time of the fitting process. The average iterative times can decrease about 50% in the experiments. This means the computational cost of the proposed model only needs half the time compared with the improved ASM. We list the average iterative times in Table 5 for each group of experiment. Of course, it can also reduce the multi-resolution levels without significant loss in accuracy for the result. Some typical results are illustrated in Fig. 9.

**Table 5:** Iterative times comparison between an improved ASM and our model.

Group index	Iterative times [16]	Iterative times (our model)
1	120.2	63.1
2	104.7	60.2
3	114.0	58.7
4	98.6	50.8
5	107.5	52.9

## Conclusions

This paper proposed a method of a deformable model utilizing local invariant features for accurate and rapid registration of cardiac images. The highly-repeatable and highly-robust local invariant features are chosen from a training set to develop a KFM. The the relationship between the KFM and the PDM is obtained statistically. During the fitting process, the PDM is transformed to its initial position according to the transformed parameters, by aligning the accurately matched SIFT features between the KFM and new images. The precise position of the PDM is further achieved by modifying the landmark coordinates in the PDM according to the relationship between the KFM and the PDM. Finally, the ultimate location of the PDM is obtained by an iterative process with less subsequent reduction in computational load.

Experiments demonstrate that this model outperforms traditional SIFT, in the sense of feature matching accuracy and robustness in cardiac MR images. Furthermore, it also surpasses other ASM regarding fitting accuracy and speed. Our experiments demonstrate that the proposed model improves the fitting accuracy by approximately 6–8% with only half of the computational cost of a state-of-the-art ASM.
